# Items

**Published:** 1883-05

**Authors:** 


### Items.

The Twenty-fourth Annual Commencement Exercises of Chicago Medical College were held at the Grand Opera House Tuesday afternoon, March 27, 1883.

The exercises included the Secretary's report of the year's work, the announcement of prizes, the conferring of degrees and charge to the class by Rev. Joseph Cummings, President of the University, and a valedictory address for the class by Dr. Geo. W. Post. The programme was pleasantly interspersed with music, and was attentively appreciated by a large audience.

Ten prizes for excellence in several different senior branches of instruction were awarded to Doctors N. S. Davis, Jr., E. G. Exler, J. E. Henderson, G. W. Post, J. T. McAnally, F. P. Peck, Chas. Davison, H. E. Burbank, James Mills and W. H. Graves.

Four prizes for excellence in anatomy and anatomical work were awarded, respectively, to Messrs. Wm. Elliot, Theo. H. Swayne, H. S. Frothingham and S. P. Black.

Dr. N. S. Davis, Jr., received the Alumni Prize for the best average scholarship in all studies, and Mr. A. C. Helm received the Edwards' Prize for best average scholarship in Junior and Middle studies. The Bullock and Grunow Prize for best examination in microscopy was awarded Dr. E. G. Exler.

The customary banquet to the graduating class and alumni was given at the Leland Hotel in the evening. It was well attended and highly enjoyed by all present.

---

DEATH OF THE SURGEON GENERAL.—Order From the General of the Army on the death of Dr. Joseph K. Barnes.

The following order was issued from the head-quarters of the army, Adjutant General's office, Washington, April 5:

Brevet Major General Joseph K. Barnes, Brigadier General of the U. S. Army (retired), late Surgeon General of the Army, died at his residence in Washington at 2 o'clock this morning. He entered the service as Assistant Surgeon, June 15, 1840; was promoted Surgeon, with the rank of Major, on August 29, 1856; Medical Inspector, with the rank of Lieutenant Colonel, February 9, 1863; Medical Inspector General, with the rank of Colonel, August 10, 1863; and Surgeon General with the rank of Bragadier General, on August 24, 1864. He was retired from active service by the operation of the law of June 30, 1882. He served with distinction in the Florida war against the Seminole Indians, in the war with Mexico, and in the war with the States in rebellion. For faithful, meritorious and distinguished services in this last war, the brevets of Brigadier General and Major General of the United States Army were conferred upon him.

He was eminent, skillful and successful in his profession as surgeon and physician, and distinguished for great administrative ability, as the head of the Medical Department. He inaugurated the medical history of the war. He founded the Medical Museum and brought the Medical Department to the highest state efficiency. During the troublous times of the late war he earned the unbounded confidence of the Secretary of War, Mr. Stanton, and held it unbroken to the last. At the time of the assassination of President Lincoln and the attempted assassination of Secretary Seward, he attended the death-bed of the one and ministered with untiring energy and skill to the successful restoration of the other. So during the long illness of President Garfield, he was one of the distinguished surgeons of the land, who for days and nights served with devoted duty in the sick chamber of the dying President. During these long protracted hours of anxiety and care his own health gave way, and from that moment to the time of his death he was an invalid. His career was one of honor to himself, and of great service to his country.

By command of General Sherman.

R. C. DRUM, Adjutant General.

---

PROFESSOR HUXLEY has been appointed the Rede lecturer at the University of Cambridge for the ensuing year.—*Medical and Surgical Reporter.*

### How They Make "Cod-Oil" at Swampscott.

The Edinburg Medical Journal, February, 1883, thus describes one of the important though unsavory interests of a neighboring watering place. Swampscott is a little town upon the coast of Massachusetts, not far from Lynn, situated near the head of a bay between Nahant and Salem, off this ancient haunt of fishermen, at a distance of about nine miles.

It is a place called the "Rocks" where in winter, the codfish come in shoals to spawn, and the striped bass sport themselves in summer. During the winter months, be the weather what it may, unless the wind be rising for a gale, a little after midnight men may be seen going about the village, stopping here and there at houses, rousing the fishermen, who, by and by gather in groups about the shore, each with his "dory," that well-known model of Yankee ingenuity which at the great Berlin fishery exhibition excited so much attention. The dories and their owners are soon aboard the various schooners in waiting, and by five A. M., the fleet is at the "rocks;" so, when the day-light is sufficient, the the dories anchor about their respective larger craft, each boat with its single occupant, who is soon hard at work robbing the sea of its life. About three P. M. the signal is given from the schooner to come aboard; the dories hasten to their floating castles, with pitchforks the various "catches" are soon thrown aboard, and sail is made for home. During the passage the fish are gutted, the entrails cast into the sea, and the livers, some of them large enough to fill a quart mug, are put into baskets. When the shore is close at hand, the fish are again put into the dories; but the roughness of the sea is such that these boats, when loaded, cannot land, and into the icy sea-water the horses are driven until the carts reach such a place that the codfish can be put into them, when off they go, to plod the night through for the early Boston market. The livers are immediately sorted over and the gall bladders carefully removed. The great luscious flabby masses are thrown into a large oak tub; with this are connected steam pipes. When the receptacle is full and closed, low pressure steam is turned on, and for about two hours and a half cooking goes on. Then the plugs are taken out at the bottom, and the hot oil streams into buckets. It is now placed in butts in the cooling-room, and allowed to stay there until it freezes solid. So it is kept until opportunity offers, when it is put into canvas bags holding about four gallons each. These bags are then placed regularly upon a heavy oak table provided with outer grooves for conducting liquid, until twelve gallons are in a row. On this is laid a slab, then canvas bags, and so layer after layer, until about eighty gallons are piled up. A ton of pig iron is then placed upon the top slab of oak and the oil begins to flow out. In about twelve hours dripping ceases, and the apparatus is taken apart. Inside the bags is found a yellowish butter-like mass as hard as tallow, which is nearly pure stearin, with liver debris and fibers. This goes to the soap makers while the oil finds its way to the Massachusetts general hospital and other places where the superiority of the finest American oil over the Norwegian is recognized.—*Boston Medical and Surgical Journal.*

---

COOK COUNTY HOSPITAL.

The following gentlemen began their service as attending physicians April 1st:

Surgeons—Drs. Hutchinson and Whitney.

Physicians—Drs. McWilliams and Buchan.

Obstetrician and Gynæcologist—Dr. DeLaskie Miller.

Ophthalmologist and Aurist—Dr. Jacobson.

Doctors Shaver and Wood began their duties as Internes. Doctors Fordyce and Hammon finished their terms as House Physician and Surgeon respectively; Dr. Fordyce commences practice at Hot Springs, Arkansas; Dr. Hammon remains in Chicago.

Hospital Clinics are as follows:

Monday, 2 p. m.—Pathology, Dr. Fenger.

Tuesday, 1:30 p. m.—Medicine, Dr. McWilliams; 2:30 p. m., Surgery, Dr. Whitney.

Friday 2:30 p. m.—Surgery, Dr. Hutchinson.

The new pavilions adjoining the present buildings are roofed, and expected to be ready for occupancy in May. The plan of their construction is essentially that of those now occupied.

Cases of pneumonia and erysipelas have been abundant during the spring; there has been no hospital epidemic.

The examination for graduation of the Training School for Nurses will be held April 19th. It will be written, and not a public occasion.

The list of examiners is not yet complete, but invitations to examine have been sent to men of acknowledged ability. The demand for nurses exceeds the supply, and but few will be available for duty.

